# Role of interferon gamma in SARS-CoV-2-positive patients with parasitic infections

**DOI:** 10.1186/s13099-021-00427-3

**Published:** 2021-05-04

**Authors:** Enas Fakhry Abdel-Hamed, Mohamed N. Ibrahim, Nahed E. Mostafa, Howayda S. F. Moawad, Nahla E. Elgammal, Ehab M. Darwiesh, Dina S. El-rafey, Nissreen E. ElBadawy, Emad Ali Al-Khoufi, Salwa I. Hindawi

**Affiliations:** 1 Medical Parasitology Department, Faculty of Medicine, Zagazig University, El Kawmia Square, Zagazig, 44511 Sharkia Governorate Egypt; 2 Clinical Laboratories Department, College of Applied Medical Sciences, Jouf University, Al-Jouf, 77451 Saudi Arabia; 3 Tropical Medicine Department, Faculty of Medicine, Zagazig University, Zagazig, 44511 Egypt; 4 Community, Environmental and Occupational Medicine Department, Faculty of Medicine, Zagazig University, Zagazig, 44511 Egypt; 5 Microbiology Department, Faculty of Medicine, Zagazig University, Zagazig, 44511 Egypt; 6 Internal Medicine Department, College of Medicine, King Faisal University, 31982, Al-Ahsa, Saudi Arabia; 7 Haematology and Transfusion Medicine, King Abdulaziz University, Jeddah, 21577 Saudi Arabia

## Abstract

**Background:**

By 27 June 2020, almost half a million people had died due to COVID-19 infections. The susceptibility and severity of infection vary significantly across nations. The contribution of chronic viral and parasitic infections to immune homeostasis remains a concern. By investigating the role of interferon (IFN)-γ, we conducted this study to understand the connection between the decrease in numbers and severity of COVID-19 cases within parasitic endemic regions. Our research included 375 patients referred to hospitals for diagnosis of COVID-19 infection. Patients were subjected to full investigations, in particular severe acute respiratory syndrome coronavirus-2 nucleic acid and *Toxoplasma* IgM and IgG antibody detection, stool examination, and quantitative IFN-γ measurement.

**Results:**

The majority of the studied cases had chest manifestation either alone (54.7%) or in association with gastrointestinal (GIT) manifestations (19.7%), whereas 25.6% had GIT symptoms. We reported parasitic infections in 72.8% of mild COVID-19 cases and 20.7% of severe cases. *Toxoplasma gondii*, *Cryptosporidium, Blastocyst,* and *Giardia* were the most common parasitic infections among the COVID-19 cases studied.

**Conclusion:**

The remarkable adaptation of human immune response to COVID-19 infection by parasitic infections with high levels of IFN-γ was observed in moderate cases compared with low levels in extreme cases. The potential therapeutic efforts aimed at the role of parasitic infection in immune system modulation are needed if this hypothesis is confirmed.

## Introduction

Severe acute respiratory syndrome coronavirus-2 (SARS-CoV-2) is an intercontinental pandemic triggered by the universal human-to-human transmission of the virus [1]. Meaningful variations are observed in the way COVID-19 spreads across different regions on various continents [2].

Malaria-endemic countries have reported a low number of COVID-19 cases [3]. The populations in these areas have natural protection against malaria infection by producing a combination of Th1 and Th2 responses. In several coinfections, malaria-induced immunosuppression also severely inhibits immune responses to other infections, such as *Salmonella* spp. [4]. By inhibiting the activity of CD8+ T-cell migration, *Plasmodium* infection protects against the *Chikungunya* virus [5].

*Toxoplasma gondii* is an intracellular parasite that generates dense granuleprotein-7 (GRA-7) into the host cell, which inhibits viral replication [6]. Based on the antagonistic phenomenon in which certain pathogens can inhibit another infection by limiting its invasion, survival, and reproduction, various parasites have weakened the severity of viral infections [7]. In addition, the concurrent infection of *Giardia* and *Rotavirus* reduces the severity of the related viral infection [8].

The most frequent symptoms of patients infected with SARS-CoV-2 upon admission are history of fever, shortness of breath, cough, fatigue/malaise, and confusion, according to the Center for Disease Control and Prevention [9]. The pathogenesis of human infection with SARS-CoV-2 manifests as mild symptoms to extreme respiratory failure. The virus replicates and migrates across the airways upon binding to epithelial cells of the respiratory tract. The rapid replication of SARS-CoV-2 in the lungs elicits an effective immune response. After the relief of clinical symptoms and negative reverse-transcriptase polymerase chain reaction (RT-PCR) assay findings, this viral infection displays an inflammatory response resulting in lung fibrosis [10].

Cytokine storm syndrome, which is considered the primary cause of death in patients, induces acute respiratory distress syndrome and respiratory failure. The serum of hospitalized patients with severe COVID-19 contain elevated levels of tumor necrosis factor, interleukin (IL)-2, IL-10, IL-7, granulocyte colony-stimulating factor, C-X-C motif chemokine ligand 10, and macrophage inflammatory protein 1α [11]. Interferons (IFNs) are well-known cytokines for their antiviral effects; they play a critical role in viral multiplication drop off [12]. The expression of IFN-γ receptors is limited to epithelial cell-enriched tissues, such as the lungs, skin, gastrointestinal tract (GIT), and respiratory tract. This receptor allocation pattern renders IFN-γ with the capability to target specific tissues [13]. The IFN-γ levels are negatively related to the increase in the amount of COVID-19 fibrosis at discharge. These results indicate the early interference of IFN-γ antiviral infection and inhibition of fibrosis for improved recovery [14].

We conducted this study to realize the relationship between the decrease in numbers and severity of COVID-19 cases within parasitic endemic areas by investigating the role of IFN-γ.

## Results

This research included 375 patients diagnosed with COVID-19 positive (+ ve) via PCR tests. The patients were aged18–69 years old, with a mean age of 44.72 years, and of which 65.6% were male, and 34.4% were female. Employers (24.8%) and specialists (18.4%) were the most frequent occupations among the studied population, as shown in Table 1. In terms of comorbidity, 40% of patients had other systemic disorders, with chronic lung disease (19.7%) being the most common. The majority of cases examined had chest manifestations either alone (54.7%) or in association with GIT manifestations (19.7%), whereas only GIT symptoms were present in 25.6%. In 92.3% of the studied cases, the severity of COVID-19 was mild, and a severe condition was noted in 7.7% (required intensive care unit (ICU) admission) (Table 1).

**Table 1:** Characters of SARS-CoV-2 +ve patients

Characteristics	(n = 375)	
	N	%
Sex		
Male	246	65.6
Female	129	34.4
Age		
Mean ± SD	44.72 ± 13.82	
Range	18–69	
Occupation		
Student	29	7.7
Unemployed house wife	51	13.6
Retired	24	6.4
Farmer	12	3.2
Skilled or worker	65	17.3
Employer	93	24.8
Specialist	69	18.4
Health care worker	32	8.5
Chronic comorbidities		
No	225	60
Yes	150	40
Hypertension	12	3.2
Diabetes mellitus	12	3.3
Hypertension + diabetes mellitus	8	2.1
Chronic heart disease	21	5.6
Chronic lung disease	74	19.7
Chronic heart + chronic lung diseases	10	2.7
Immunosuppression	13	3.5
COVID-19 symptoms		
GIT manifestation	96	25.6
Chest and GIT manifestations	74	19.7
Covid-19 severity		
Mild	346	92.3
Severe (admission ICU)	29	7.7

**Fig. 1:**
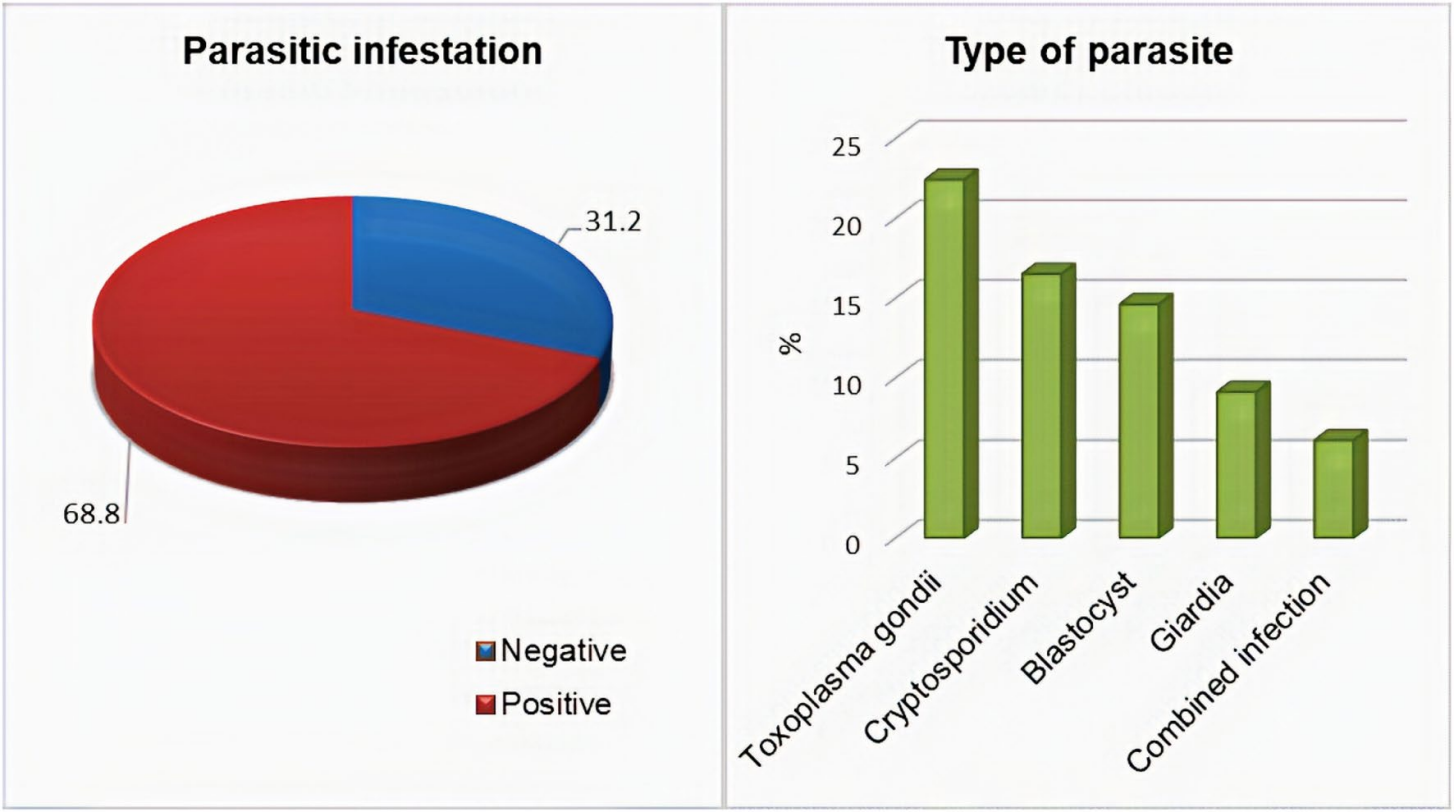
Frequency of parasitic infections and types of parasite among SARS-CoV-2 +ve patients

The prevalence of parasitic infections among the studied COVID-19 cases was 68.8%. The most commonly found parasites were *Toxoplasma gondii* (22.4%), *Cryptosporidium* (19.7%), *Blastocyst* (17.6%), *Giardia* (9.1%), and *Cryptosporidium* + *Blastocyst* + *Entamoeba histolytica* combined infection (6.1%) (Fig. 1).

Table 2 reveals the relationship between the severity of COVID-19 infection and symptoms, where all confirmed severe cases showed chest symptoms only with a highly statistically significant difference (*P* < 0.001) compared with the mild cases. The mild cases showed chest symptoms (50.9%), GIT symptoms (27.7%), and mixed chest and GIT symptoms (21.4%) with a highly statistically significant difference (*P* < 0.001). Reviewing the relation between parasitic infected cases and the severity of this viral infection, 72.8% and 20.7% of mild and severe cases had parasitic infection, respectively (Table 3). Additionally, 91.7% of cases with diabetes mellitus had parasitic infections, and 75% of hypertensive cases and 100% of immune-compromised patients had parasitic infections with a statistically significant difference (*P* < 0.05). A highly statistically significant increase was observed in the frequency of infections among cases with GIT symptoms (78.1%) and combined GIT and chest symptoms (83.8%) compared with cases with chest symptoms only (59%), indicating a high statistically significant difference (*P* < 0.001).

**Table 2:** Relation between severity of SARS-CoV-2 and manifestation

Manifestation	Mild (n = 346)		Severe (n = 29)		χ^2^	*P*
	N	%	N	%		
Chest manifestation	176	50.9	29	100	26.06	< 0.001**
GIT manifestation	96	27.7	0	0		
Chest & GIT manifestations	74	21.4	0	0		

*χ*^*2*^ Chi-square test, ** highly significant (*P*  < 0.01)

**Table 3:** Comparison between parasitic infected cases and free cases in terms of demographic data, comorbidity, SARS-CoV-2 manifestation, and severity

Characteristics	Total	Negative (−ve) (n = 117)	Positive (+ve) (n = 258)	t	*P*
Age					
Mean ± SD	375	43.37 ± 10.62	45.11 ± 12.53	1.30	0.19
Range		18–67	19–69		NS

Characteristics	Total	N	%	N	%	χ^2^	P
Sex							
Male	246	75	30.5	171	69.5	0.17	0.68
Female	129	42	32.6	87	67.3		NS
Occupation							
Student	29	11	37.9	18	62.1		
Unemployed-housewife	51	13	25.5	38	74.5		
Retired	24	10	41.7	14	58.3		
Farmer	12	1	8.3	11	91.7		
Skilled or worker	65	23	35.4	42	64.6	14.98	0.04*
Employer	93	35	37.6	58	62.4		
Specialist	69	21	30.4	48	69.6		
Health care worker	32	3	9.4	29	90.6		
Chronic comorbidities							
No	225	69	30.7	156	69.3	0.07	0.78
Yes	150	48	32	102	68	0.07	NS
Hypertension	12	5	41.7	7	58.3		
Diabetes mellitus	12	1	8.3	11	91.7		
HPT + DM	8	2	25	6	75	12.58	0.04*
Chronic heart disease	21	9	42.9	12	57.1		
Chronic lung disease	74	26	35.1	48	64.9		
Chronic heart & lung diseases	10	5	50	5	50		
Immunosuppression	13	0	0	13	100		
COVID-19 symptoms							
Chest manifestation	205	84	41	121	59		
GIT manifestation	96	21	21.9	75	78.1	20.67	<0.001**
Chest & GIT manifestations	74	12	16.2	62	83.8		
Mild	346	94	27.2	252	72.8	33.89	<0.001**
Covid-19 severity							
Severe (admission ICU)	29	23	79.3	6	20.7		

*SD* standard deviation, *t* independent t test, *χ2* chi-square test, *NS* non-significant, *NS* non-significant (*P * >  0.05) * significant (*P*  <  0.05) ** highly significant (*P*  <  0.01)

The parasitic infection modulated the human immune response to COVID-19 infection in mild cases with high levels of IFN (50.17 ± 8.24) compared with those with low levels of IFN (9.17 ± 1.60) in severe cases (Table 4).

**Table 4:** Relation between IFN-γ and the severity of SARS-CoV-2 with and without parasitic infection

IFN-γ level (pg/ml)	Total (375)	Mild cases (n = 346)		Sever cases (n = 29)		F	*P*
		With parasites (n = 252)	No parasites (n = 94)	With parasites (n = 23)	No parasites (n = 6)		
Mean ± SD	40.29 ± 16.94	50.17 ± 8.24 a	25.49 ± 5.92 b	9.17 ± 1.60 d	0.74 ± 0.22 c	529	< 0.001**

*SD* standard deviation, *F* ANOVA test, ** highly significant (*P*  <  0.01)

Groups with different letters are statistically significant (*P*  <  0.05)

## Discussion

Our results reported the decreased incidence and severity of SARS-CoV-2 disease in patients with parasitic infections compared with those who had SARS-CoV-2 alone. Among the examined COVID-19 cases, the prevalence of parasitic infections was 68.8% with a highly statistically significant increase (*P* < 0.001) in mild cases (72.8%) compared with that in severe cases (20.7%). The most commonly identified parasites (Fig. 1) were *Toxoplasma gondii* (22.4%), *Cryptosporidium* (19.7%), *Blastocyst* (17.6%), and *Giardia* (9.1%).

In our research, the path of the disease was mild given that spontaneous resolution occurred in *Toxoplasma gondii*-infected patients. These results can be explained by the production of GRA-7, which displays immune stimulation and a broad spectrum of antiviral activities via type I IFNs signaling [6]**.** Another clarification was obtained by Degrandi et al. [15]**,** who announced that *Toxoplasma*-infected mice exhibited up-regulated immune responsive gene 1 in their lungs. This gene is an IFN-stimulated gene that mediates antiviral effects against RNA viruses [16]. Moreover, the apicoplast proteins of protozoa have immunogenic potential [17]. Priming with viable *C. parvum* oocysts provides a protective immunity at an extremely low dose. This condition restores the Th1-partner mucosal (CD8+ T-cells) and their cytokine IFN-γ effectors that otherwise decrease with ongoing protein malnutrition [18]. Nevertheless, after respiratory syncytial virus, *Heligmosomoide spolygyrus* considerably reduces pulmonary complications [19]**.**

In our study, we obtained high levels of IFN-γ (50.13 pg/ml) in mild cases compared with the low levels (0.7385 pg/ml) in severe cases of COVID-19 with parasitic infections. Chen et al. [10], who reported that IFN-γ was lower in severe COVID-19 patients compared with those with a moderate form of the disease, approved our findings. Bajwa et al. [20] also noted the antiviral activity of IFN-γ by exerting cellular effects at various levels. IFN-γ interacts with the viral receptor, resulting in the consequent reduction of several virus replication down-regulating genes and gene products [21]. Li and De Clercq [22] showed that IFNs are potential drug choices for SARS-CoV-2 infection.

Our main finding supports the protective role of parasitic infections against infection with COVID-19. Compared with parasite-free patients, the general improvement in the overall health status of COVID-19 +ve patients was demonstrated by a rapid exit without any respiratory complications. Our findings are endorsed by Cohen et al.[23], who stated that CD4+ and CD8+ T-cells play imperative synergistic roles given that both secrete IFN-γ during *T. gondii* infection. The IFN-γ produced by CD4+ cells is required to prime macrophages, inducing powerful antimicrobial responses. IFN-γ regulates the expression of inducible nitric oxide synthase, which is eventually required for parasite control [24]. Additionally, treatment with soluble *T. gondii* antigen 24 h post-*P.berghei* infection resulted in a rapid increase in serum IL-12 and IFN-γ levels [25]. The development of immunity in *Cryptosporidium*-infected humans and murines is associated with the elevated expression of IFN-γ Th1 cytokines [26]. In addition, the immunomodulation caused by malaria is effective against extreme manifestations of certain respiratory viruses. Hospitalized children diagnosed with influenza and malaria in Kenya are less likely to suffer respiratory distress than those with influenza alone [27].

## Conclusion

Among the parasite-infected cases, the COVID-19 disease sequence was moderate. *Cryptosporidium, T. gondii, Blastocyst*, and *Giardia* were parasitic infections among the studied COVID-19 infected cases. A high percentage of moderate COVID cases accompanied with parasitic infections was observed in our results, and an increased number of serious cases were parasite free. The +ve role of parasitic infections in modulating the human immune response to COVID-19 infection was evident in the high level of IFN γ in mild cases. A new prospect for the development of novel successful protection against COVID-19 infection has been provided by the unclear link between parasitic and viral infections. Testing the safety and efficacy of parasite-derived antigens in more patients with SARS-CoV-2 infection may be worthwhile. Our future research will focus on the cytokine storm concerning parasitic infections triggering the immunocompromised population.

## Material and methods

### Population study

This cross-sectional analysis was conducted on 375 patients who were referred to Zagazig University Hospital and Al-Ahrar Hospital in Sharkia Province, Egypt for diagnosis of COVID-19 infection between 15 July and 10 October 2020. The requirements for diagnosis of the suspected cases of infection were sent to the Egyptian Ministry of Health Protocol for Diagnosing and Treating Cases of COVID-19. The reported patient had a strong history of epidemic exposure and had at least two of the following clinical manifestations: fever, respiratory symptoms, viral pneumonia imaging, and normal or decreased number of white blood cells and lymphocytes at the early stage of the disease. Complete blood count, SARS-CoV-2 nucleic acid detection, lung computed tomography analysis, specific *Toxoplasma* IgM and IgG antibody detection, and stool examination were conducted onall patients involved. No exclusion criteria were applied for the patients. The cases examined were subjected to medical history and clinical examination and then divided into those with mild disease with fever, fatigue, dry cough, and ground-glass opacities of pneumonia. The total number of white blood cells in the early stage of disease was normal or decreased, the lymphocyte count decreased, and severe disease occurred with respiratory distress (respiration rate ≥ 30 times/min), oxygen saturation (≤ 93% in the resting state), PaO_2_/FiO_2_ ≤ 300 mm Hg. Upon admission, blood samples were obtained from the patients. Nine days after symptoms appeared, the medication regimen was started. Mild cases appeared after a week. On the other hand, in severe cases, oral intubation was conducted in ICU during treatment.

### Diagnosis of COVID-19 infection

SARS-CoV-2 RNA was investigated by RT-PCR of the combined nasopharyngeal and oropharyngeal swab samples of the reported cases of mild and serious infection, as per the Egyptian Ministry of Health diagnostic guideline.

Viral RNA was extracted by using the automated extraction system GenoXtract® from HAIN Lifescience GmbH (Brucker) using GXT DNA/RNA Extraction kit (CE IVD) (Primerdesign Ltd, School Lane, Chandler’s Ford, UK, SO53 4DG). RT-PCR was performed with genesig® Real-Time PCR Coronavirus (COVID-19) (CE) Z-Path-COVID-19-CE- (Primerdesign Ltd, School Lane, Chandler’s Ford, UK, SO53 4DG) using primers and probes targeting the RNA-dependent RNA polymerase gene fragment in Roche® LightCycler 480 II. Each 12 µL reaction mixture contained 10 µL oasig qPCR OneStep Master Mix and 2 µL Coronavirus (COVID-19) CE IVD Primer/Probe. The thermal cycling conditionswere10 min at 55 °C for RT, 2 min at 95 °C for initial PCR activation, and 45 cycles of 10 sat 95 °C and 60 sat 60 °C, in accordance with the manufacturer’s instructions (Primerdesign Ltd, School Lane, Chandler’s Ford, UK, SO53 4DG). The +ve control template contained standardized concentrations of SARS-CoV-2 RNA (COVID-19) specific sequence at a concentration of 1.67 × 10^5^ copies per µl. The template should produce Cq ≤ 22 in the FAM channel to ensure the PCR run validity. Each run should contain the +ve and the negative control to produce a valid result. Analytical and clinical performances of the kit were determined by the manufacturer’s instructions.

### Parasitological assessment

#### Diagnosis of toxoplasmosis

Venous blood (approximately 5 ml) was collected from each patient. The blood samples were labeled and refrigerated by shipment to the laboratory at 4 °C. The blood samples were then centrifuged in 1.5 ml centrifuge tubes to obtain the serum. The samples were held at − 80 °C before use. The electrochemiluminescence immunoassay (Cobas E411 immunoassay analyzers, Mannheim, Germany) was used to detect anti-*Toxoplasma* IgG and IgM antibodies. Master curve minimum and maximum limits specified by 0.13–650 IU/ml were detected for reference measurement.

#### Stool examination

Fresh stool samples from patients with manifestations of COVID-19 were collected. Direct smear, formol–ether concentration technique [28]**,** and modified Ziehl–Neelsen stain [29] were performed. On the basis of GIT manifestation of most patients, parasitological evaluation was requested.

### IFN-γ measurement

The human IFN γ enzyme-linked immunosorbent assay kit (NOVA, No. 18, Keyuan Road, DaXing Industry Region, Beijing, China) was used to quantitatively calculate IFN-γ in the serum of the studied population, following the manufacturer’s instructions. Optical density was estimated at 450 nm, the standard curve was built, and the mean absorbance concentration of the samples from the standard curve was measured.

### Statistical analysis

The data were computerized and statistically analyzed using Statistical Package for the Social Sciences, version 25.0. Qualitative data were expressed as the number and percentage, and quantitative data were expressed as the mean, standard deviation, and range. Chi-square (χ^2^) and independent t-tests were used in comparing data. The p-value was considered significant if < 0.05 and highly significant if < 0.01.
